# Physiological Traits for Predicting Poleward Extensions in Tropical Fishes: From Lab to Management

**DOI:** 10.1111/gcb.70213

**Published:** 2025-04-28

**Authors:** Adam T. Downie, Curtis Champion, David J. Booth

**Affiliations:** ^1^ School of Mathematical Sciences Queensland University of Technology Brisbane Queensland Australia; ^2^ Fisheries Research, Department of Primary Industries and Regional Development National Marine Science Center Coffs Harbor New South Wales Australia; ^3^ School of Life Sciences University of Technology Sydney Sydney New South Wales Australia

**Keywords:** adaptation, climate change, habitat shifts, range shifts, species‐on‐the‐move, tropicalization

## Abstract

Tropicalization, the phenomena by which tropical organisms are extending their distributions poleward into temperate latitudes in response to increasing temperatures and strengthening boundary currents, is occurring globally. Vagrant tropical species have large ecological and economic ramifications for the temperate habitats they invade. However, not all vagrants are able to persist long term in temperate habitats, with the first winter being a potential bottleneck for their persistence. This brings into question how some tropical vagrant species are successful at surviving temperate conditions and the physiology underpinning this success. This provides the opportunity to not only look at the available data introspectively but also forward‐thinking by applying a range of holistic physiological traits relevant for biology and management. Therefore, the aim of our review is twofold: to review the current state‐of‐knowledge of the physiological mechanisms underpinning tropicalization and to develop a physiological framework by which current practices can complement new perspectives and tools. We use range‐expanding tropical reef fishes as a model group of over 100 species undergoing climate‐driven range shifts and eastern Australia as a case‐study location due to it being a primary focal “living laboratory” for understanding tropicalization dynamics since the early 2000s. Current studies suggest that diet, behavior, and metabolic trade‐offs may explain vagrant fish persistence, but these studies focus on whole‐animal traits. Our framework helps expand upon focal traits, life stages, experimental design, physiological traits (e.g., we highlight the value of genetic and cellular markers for metabolic pathway changes under cold stress as potential biomarkers) and species to improve our understanding of the mechanisms underpinning tropicalization. Taken together, our framework places emphasis on measuring a suite of complimentary physiological traits, from cellular to whole‐animal, to help guide future predictions of the long‐term persistence of tropical species in temperate habitats.

## Graphical abstract

We review the physiological mechanisms that permit poleward range expansions of tropical reef fishes into temperate waters. Filling in key knowledge gaps, we develop a framework to improve the implementation of physiological traits in improving our understanding of how tropical species adapt to temperate habitats.

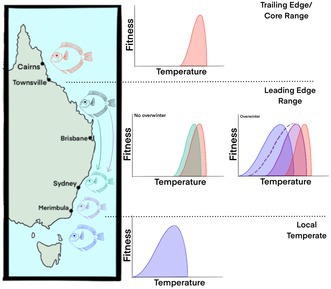

## 1 Introduction

The effects of human‐induced climate change are causing large‐scale impacts that will have long‐term consequences for biodiversity and ecosystem function and services (Hughes et al. 2003, 2017). In response, science can support the management of changing ecosystems by improving our predictive capacity of population responses to change (either through evolutionary adaptation or phenotypic plasticity) and improved understanding of the consequences of selection pressure from environmental change on certain traits and species above others (Thoral et al. 2024). Specifically, the sub‐discipline of “Conservation Physiology” can support the science‐based management of climate impacts on marine systems through its fundamental understanding of organismal physiological response to environmental change based on a range of well‐established methodologies that can directly link organism health and function with environmental conditions, such as measurements from enzyme function to whole‐animal growth (Madliger et al. 2018; Wikelski and Cooke 2006). Indeed, the measurement of a suite of synergetic physiological traits, from cell to whole animal, can therefore be used as valuable biomarkers to assess species as they undergo redistributions with shifting environmental gradients (Adams and Ham 2011).

Climate‐driven range shifts are one of the most evident biological responses to climate change, particularly in marine systems where rates of redistributions markedly exceed those of terrestrial systems (Lenoir et al. 2020; Pecl et al. 2017). As species redistributions occur in response to environmental change, plants and animals seek new refuge areas at higher latitudes and higher altitudes (terrestrial habitats) and deeper depths (aquatic habitats) that are within their physiological performance and tolerance limits (Chen et al. 2011; Pecl et al. 2017; Sunday et al. 2012). Specifically, animals and plants inhabiting low latitudes (i.e., tropics) generally live within narrow seasonal temperature ranges that approach their upper thermal tolerance limits (Rummer et al. 2014). Therefore, small increases in temperature have large consequences on tropical species' physiology, survival, and capacity to adapt under such conditions (Habary et al. 2017; Higgins et al. 2024). In response, tropical and sub‐tropical species populations that are unable to adapt to warming in situ conditions, either through plasticity in thermal tolerance limits or shifting to deeper depths, are moving on average 60 km decade^−1^ away from the equator to seek refuge in habitats situated poleward; a phenomenon known as tropicalization (Chen et al. 2011; Gervais et al. 2021; Lenoir et al. 2020; Pecl et al. 2017; Pinsky et al. 2020). Successful invasions of tropical vagrants into some temperate habitats have had devastating economic and ecological effects on global ecosystems (Zarzyczny et al. 2024), with notable examples from Australia (Vergés et al. 2019; Vergés, Steinberg, et al. 2014; Vergés, Tomas, et al. 2014; Wernberg et al. 2016), North America (Osland et al. 2021), and Europe (Chust et al. 2024). Therefore, from a management perspective, predicting which tropical species will move and be successful in temperate habitats will be critical to understanding population dynamics and potential changes in ecosystem function and services with the presence of tropical vagrants.

Predicting range shifts of tropical species into temperate waters is imperative for local ecosystem management and persistence of temperate habitats into the future (Bonebrake et al. 2018; Pecl et al. 2017; Vergés et al. 2019). While correlative models are very useful in mapping species distributions in relation to climate, they do not verify the mechanisms that limit or expand species' ranges (Buckley 2008; Kearney and Porter 2009). Currently, there is a need for mechanistic models parameterized and validated using biological data to help predict species range shifts in response to changing climate (Kearney and Porter 2009; Twiname et al. 2020). Mechanistic models must be accurately parameterized or else their predictive skill is considerably reduced, which presents an issue for species with limited physiological or biological information (Kearney and Porter 2009). The use of physiological traits such as thermal limits (Carbonell and Stoks 2020; Carbonell et al. 2021; Lehmann et al. 2015), and cellular and genetic markers of thermal tolerance (Lancaster et al. 2016) have been valuable tools used to predict habitat shifts in fishes and other organisms, with applications varying from estimating range shifts due to climate change (Lancaster et al. 2016) and monitoring or predicting invasive species (Kearney and Porter 2009). Additionally, taking a meta‐analytical approach, Wu and Seebacher (2022) found that individuals from populations near their leading edge (populations living in the cooler end of the species' habitat range) had increased metabolism, immunity, thermal tolerance capacity, locomotor capacity, and hormone release compared to individuals at core (populations living in the middle of the species' habitat range, where most individuals inhabit) and trailing edge (populations living in the warmest part of the species' habitat range) populations. These are all measurable traits with testable hypotheses as per an animal's physiological capacity for range expansion. Therefore, physiological metrics are valuable predictors of the capacity of species to move to and be successful in novel habitats with varying thermal regimes. Unfortunately, implementation of physiological data into management practices has been limited, due to several challenges. First, we generally measure whole‐animal traits of physiological performance (e.g., growth, locomotion, development, metabolic rate), which provide a limited perspective on organismal response to environmental change. Rarely do we consider the impact of environmental change on the whole mechanism, linking together basal physiological traits (cellular, genetic and molecular processes), and tissue and organ systems, with whole‐animal performance with the aim of understanding population dynamics (Madliger et al. 2018; McKenzie et al. 2016). This holistic view is important because basal physiological traits set the pace at which tissue and organ systems function, which directly influences the development and function of the organism. Secondly, physiological traits are rarely co‐measured or correlated with ecological processes or techniques such as in situ surveys, population dynamics modeling or movement patterns (Adams and Ham 2011; Bergman et al. 2019; Thoral et al. 2024). These first two challenges may be expensive, time consuming, resource consuming, logistically challenging, and reliant on niche expertise of techniques and processes. Thirdly, historically, physiological data are generally not made available directly to managers and policy makers and are restricted to published literature; especially in data relevant for fisheries (McKenzie et al. 2016). Regardless, these barriers should be broken down and improved access to these resources available to provide a holistic view of how environmental change impacts individual performance and species' adaptive capacity to change, which will have consequences on our understanding of critical ecosystem processes.

While there are several frameworks available for incorporating physiology into management (e.g., Brosset et al. 2021; Lagadic 2001; Madliger et al. 2018; McKenzie et al. 2016; Rossi et al. 2017; Twiname et al. 2020), there remains considerable scope for utilizing species' physiological traits to specifically predict tropicalization. This is critical given the global reach of this ecological issue, and the complexities and ramifications of better understanding how animals occupy their potential thermal niche. Understanding these challenges may provide predictive power for a suite of ecological scenarios, such as understanding the persistence of tropical vagrants in temperate habitats, which tropical species may have the capacity to shift poleward that have not already, and what are the consequences of future projected climate change on local ecosystem function and services. Here, we take a case‐study approach using ecological and physiological studies from eastern Australia; an important “living laboratory” that is of heavy research focus for tropicalization globally (Booth et al. 2018; Figueira and Booth 2010; Pecl et al. 2017). Specifically, we focus on tropical coral reef fishes inhabiting coastal Queensland that have expanded their range south into temperate New South Wales, which has been of high research interest since the early 2000s (Booth et al. 2018, 2007; Gervais et al. 2021). First, we briefly describe climate impacts on coral reef fishes to provide context as to why fish are shifting south. Then, we review the current physiological literature on studies that have investigated how tropical vagrants tolerate winter conditions in New South Wales. Finally, we develop a physiological framework consisting of a toolbox of traits, techniques and measurements that will be valuable when partnered with ecological approaches. This included a second literature search focusing on cold tolerance of sub‐tropical and tropical fishes regardless of geographic location to review traits not yet considered in tropicalization research. These traits can be used in future research initiatives to improve our understanding of the persistence of tropical species in cooler, temperate habitats. Some of these approaches (e.g., secondary stress biomarkers) have to date been applied within an aquaculture setting but can be easily translated and applied within the context of ecological monitoring and would significantly benefit our understanding of tropicalization. This framework's aim is to reduce the challenges outlined previously by taking physiological data outside the laboratory and into a broader, applicable setting. Our approach supports the increasing importance of integrating the sub‐discipline of Conservation Physiology's fundamental principles into ecosystem management, because understanding the mechanisms underlying tropical species' adaptation to temperate habitats has significant bottom‐up effects on predicting future range shifts and the ecological and economic consequences of short‐ and long‐term persistence of vagrants in temperate waters.

## 2 Literature Search

Two sets of literature searches were performed for this study. The first literature search focused on peer‐reviewed research papers on physiological responses of vagrant tropical or sub‐tropical fish species to temperate conditions in Australia (Section 4.2). We accessed Web of Science (August 2024) and used the following search term: (tropicalization OR habitat expansion OR range expansion) AND (Australia*) AND (fish* OR teleost* OR reef fish* or vagrant*) and obtained 665 results. We went through title and abstracts, with the following criteria to be included: the study had to include a vagrant tropical fish exposed to temperate water conditions under experimental settings or collected from field sampling along the east coast of Australia. We did not include studies whose winter conditions were derived from future climate projections given their associated uncertainty. Physiological metrics (e.g., growth, metabolic rate, metabolites, gene expression etc.) or associated behaviors (e.g., bite rate) had to be measured to be considered in our review. That left us with 16 papers to include. The second set of literature searches focused on peer‐reviewed research papers on physiological responses of tropical or sub‐tropical fish species to cold stress, regardless of country of origin (Section 5, step 4). We accessed Web of Science (October 2024) and used the following search term: (cold tolerance* OR thermal minima OR cold stress* OR lower thermal limits) AND (teleost* OR fish*) and obtained 374 results. We did not explicitly include “tropical” or “sub‐tropical” as search terms to avoid missing relevant papers that did not explicitly state whether the study species were tropical or sub‐tropical in the title, abstract or methods. Most of the selected studies were undertaken in Asia within the context of understanding the impacts of cold snaps of tropical species in aquaculture. We went through title and abstract, and to be considered, experimental design had to consist of a tropical or sub‐tropical fish species exposed to ambient and cold temperatures, and physiological metrics evaluated, particularly metrics not yet considered in experiments performed in tropicalization studies involving fishes (e.g., transcriptomic and metabolomic approaches to determine cold effects on metabolic pathway alterations and biomarker development). We found 30 papers appropriate for our review.

## 3 Range Extensions of Tropical Fishes Along the Eastern Australian Coast

**FIGURE 1:**
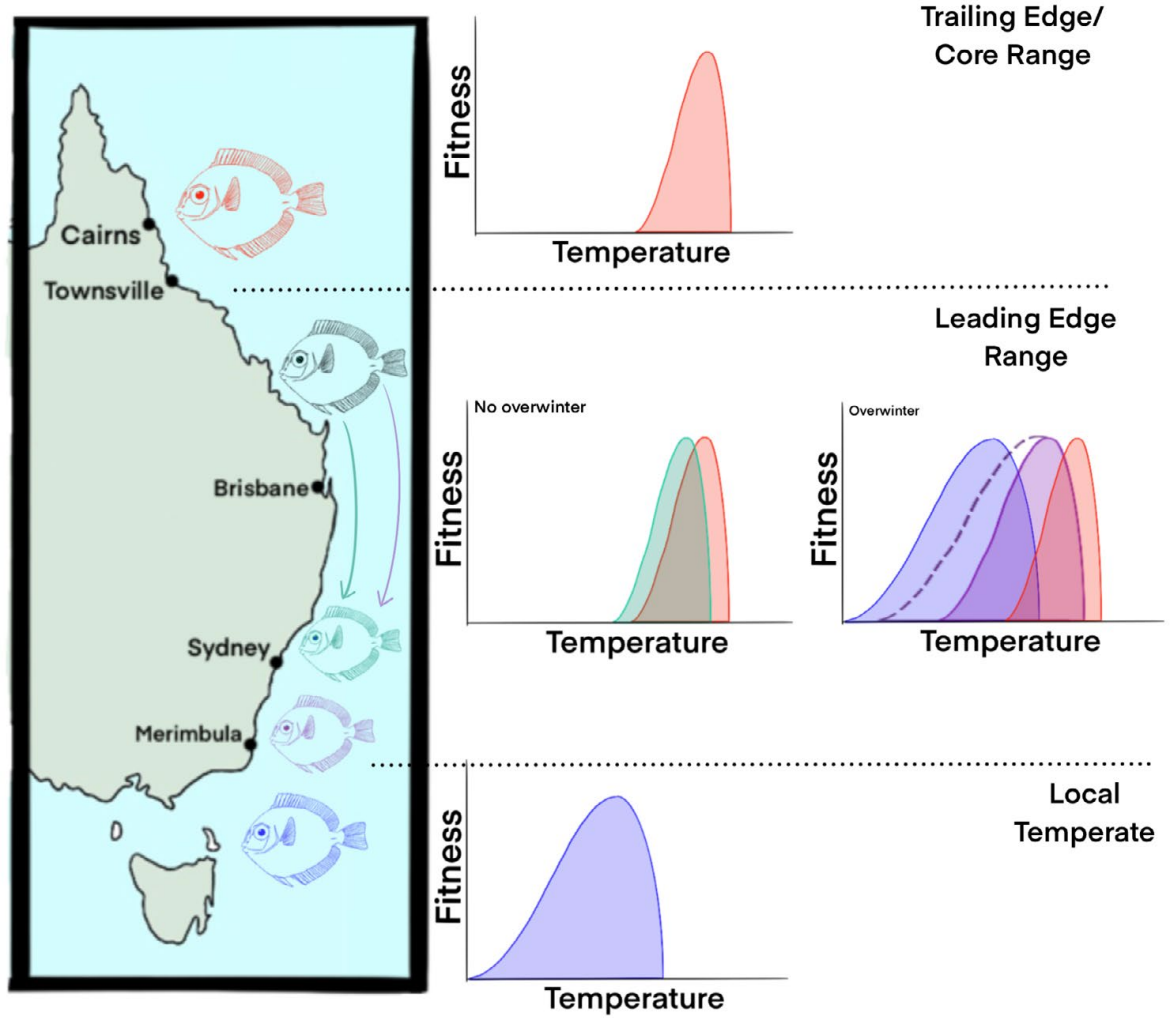
Hypotheses for the relationship between organism fitness and thermal habitat for fish populations across latitude, along the East coast of Australia. Populations inhabiting northern tropical or sub‐tropical reefs (red fish; Trailing Edge or Core Range) are exposed to a narrow seasonal thermal regime, compared to fishes naturally inhabiting temperate habitats (blue fish) that experience wider ranges in seasonal temperatures. Tropical or sub‐tropical populations on the southern edge of their habitat range (black fish; leading edge) are predicted to experience a wider range of temperatures than those within the core range or trailing edge populations. Leading edge populations are predictably moving polewards into temperate habitats. Those that migrate poleward but are unable to overwinter are hypothesized to have thermal tolerances similar to core or trailing edge range populations or traits are not plastic enough to respond to change (green fish). However, individuals that migrate south and successfully overwinter are hypothesized to have tolerances similar to temperate species (purple fish) or their tolerances are plastic to change (purple dotted line on figure). Colours of fish correlate to colours of performance curves. Map lines delineate study areas and do not necessarily depict accepted national boundaries. Fish and figure drawn using Procreate (Ver. 5.3.15) by ATD.

**FIGURE 2:**
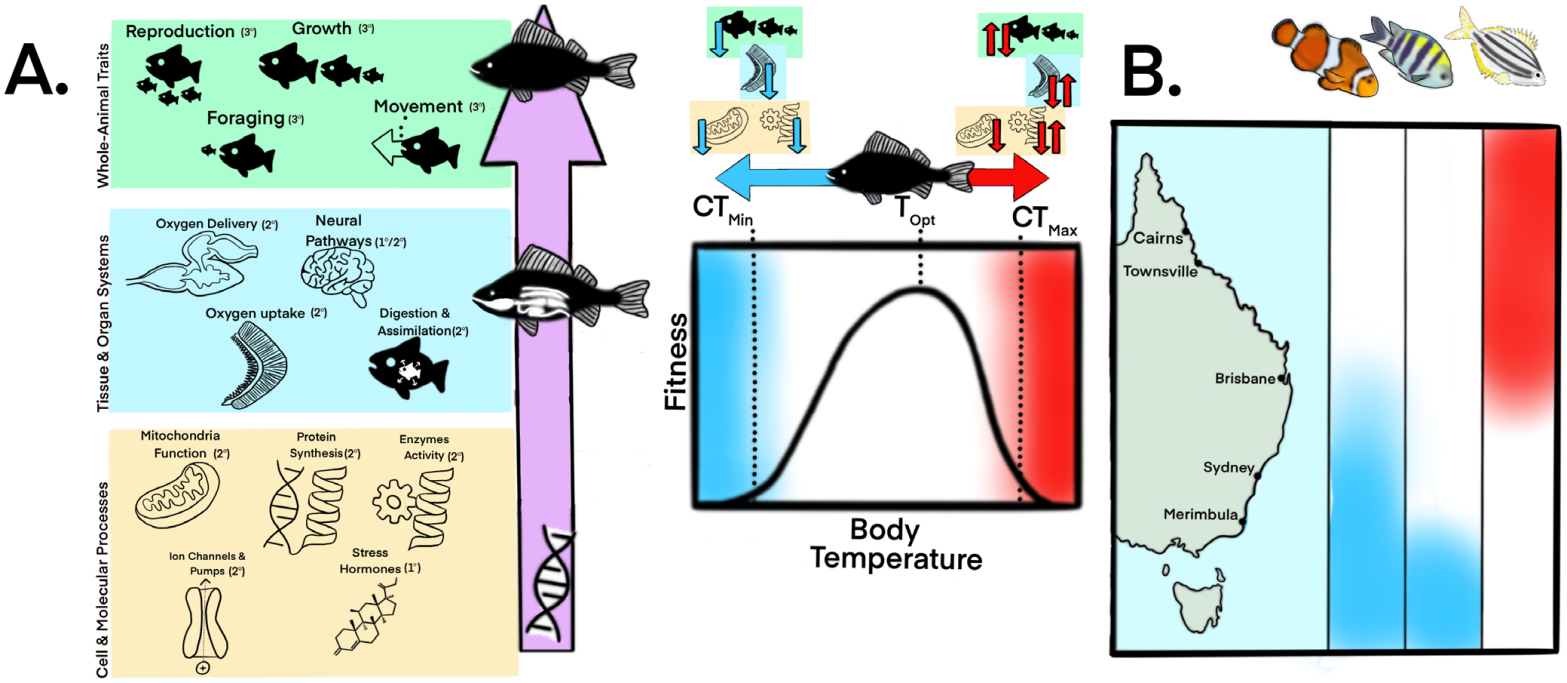
Bottom‐up effects of cellular to whole‐animal physiological traits, which dictates individual fitness, and the potential ramifications on population thermal tolerance limits and habitat ranges, using coastal marine fishes along the east coast of Australia as the case‐study group of species. (A) Thermal tolerance limits are governed by a bottom‐up cascade of thermally sensitive physiological traits from cellular, molecular, and genetic traits that set the pace of physiological reactions, which influences the rate at which tissue and organ systems operate, which in‐turn impacts the general development and function of the whole animal. Some of these traits are important measures of primary (1°), secondary (2°), and tertiary (3°) stress responses, as indicated beside each icon. Exposure of animals below thermal optima (*T*_opt_) to critical thermal minima ranges (CT_min_) generally results in reduced physiological function from cell to whole animal. Exposure of animals above *T*_opt_ (i.e., critical thermal maxima, CT_max_) typically increases the physiological function on an acute temporal scale; however, chronic stress or high temperature generally decreases physiological performance. (B) Hypothetical habitat ranges for (from left to right) tropical (orange clownfish; *Amphiprion percula* ), sub‐tropical (Sergeant Major; *Abudefduf vaigiensis* ), and temperate (Australian mado; *Atypicthys strigatus*) coastal species are represented by colored bars that correlate to the thermal performance curve. White represents preferred habitat range annually as this is within the species/populations' thermal optima (*T*_opt_). Blue represents lower thermal limits (CT_min_), and red represents upper thermal limits (CT_max_) and are areas outside of the thermal limits of the species/population and are latitudes that would have potential sub‐lethal and lethal physiological consequences. Map lines delineate study areas and do not necessarily depict accepted national boundaries. Fish and figure drawn using Procreate (Ver. 5.3.15) by ATD.

Among the fastest range shifts of tropical reef fish species are those occurring on the east coast of Australia. Australian tropical reef species are moving on average 38 km decade^−1^ south into temperate waters (Gervais et al. 2021; Sunday et al. 2011, 2012, 2013, 2015). In fact, over 100 species of tropical reef fishes (> 10 families, > 20 genera) from populations inhabiting the southern Great Barrier Reef (GBR) are shifting to temperate reefs (Booth et al. 2018, 2007; Fowler et al. 2018). Such dispersal patterns predictably occur during the pelagic larval phase for reef fishes, as adults are generally site‐attached. Such poleward movement patterns are a result of the intersection of ocean hydrodynamics and larval biology, with larvae being carried from the GBR south via increasingly strengthening and warming boundary currents such as the Eastern Australian Current (EAC; Booth et al. 2007, 2018) and the use of well‐developed swimming and sensory systems to navigate to new habitats (Downie et al. 2020; Downie, Lefevre, et al. 2023; Downie, Leis, et al. 2021; Fisher et al. 2005; Leis 2006). Such dramatic latitudinal shifts are likely linked to the differences in physiological thermal limits of individuals of species living in leading edge populations versus trailing and core edge populations of their habitat range, and tolerance limits of life‐history stages (Figures 1 and 2). The specific mechanisms are currently unknown, albeit the topic of interest in this review. For example, do newly arriving larvae/young juveniles have the biochemical capacity to alter metabolic pathways to tolerate temperate habitat conditions, or switch to alternate energy pathways to fuel organ function? These topics will be discussed in more detail in Section 5, but it is important to note early that we know very little about the physiological tolerances of newly recruiting tropical fishes to temperate reefs.

Tropical fishes naturally live within a narrow temperature range (i.e., low seasonal variation in water temperature), and many of these species live close to their maximum tolerance limits (Figueira et al. 2009; Rummer et al. 2014). Therefore, the predicted 1°C–3°C increases in ocean temperature characterizing both current summer heatwave events and the end‐of‐century projections will likely be lethal for many tropical fish species (Habary et al. 2017), forcing them to either adapt to conditions or seek deeper depths (see Section 4.1 for details) in local warming habitats or move south to seek refuge in cooler waters within their optimal thermal limits (Donelson et al. 2019). Moving to cooler, temperate waters is generally lethal for tropical species, but summertime temperatures in the southern temperate reefs in Australia (i.e., New South Wales) are considered thermal “hotspots” (Figueira et al. 2009; Fowler et al. 2018). These coastal habitats are experiencing summer warming rates that are approximately three‐times greater than the global average, creating potential refuge sites for tropical fishes (Figueira et al. 2009; Fowler et al. 2018). Despite warming conditions, winter in temperate habitats is still considered a “survival bottleneck” for tropical species, as seasonal winter temperatures below 18°C result in mortality for most individuals (Booth et al. 2018). According to Booth et al. (2018), 13% of species (20/150) species survive overwinter in Sydney (34° S) and 8% of species (5/60 species) survive further south in Merimbula (37° S). However, some tropical fish species do survive winter, such as the Sergeant Major ( *Abudefduf vaigiensis* ; Pomacentridae), Yellow‐back puller (*Chromis nitia*; Pomacentridae), Moon Wrasse ( *Thalassoma lunare* ; Labridae) (Booth et al. 2018), as well as some members of Siganidae (i.e., rabbitfishes; Vergés, Steinberg, et al. 2014; Vergés, Tomas, et al. 2014).

**FIGURE 3:**
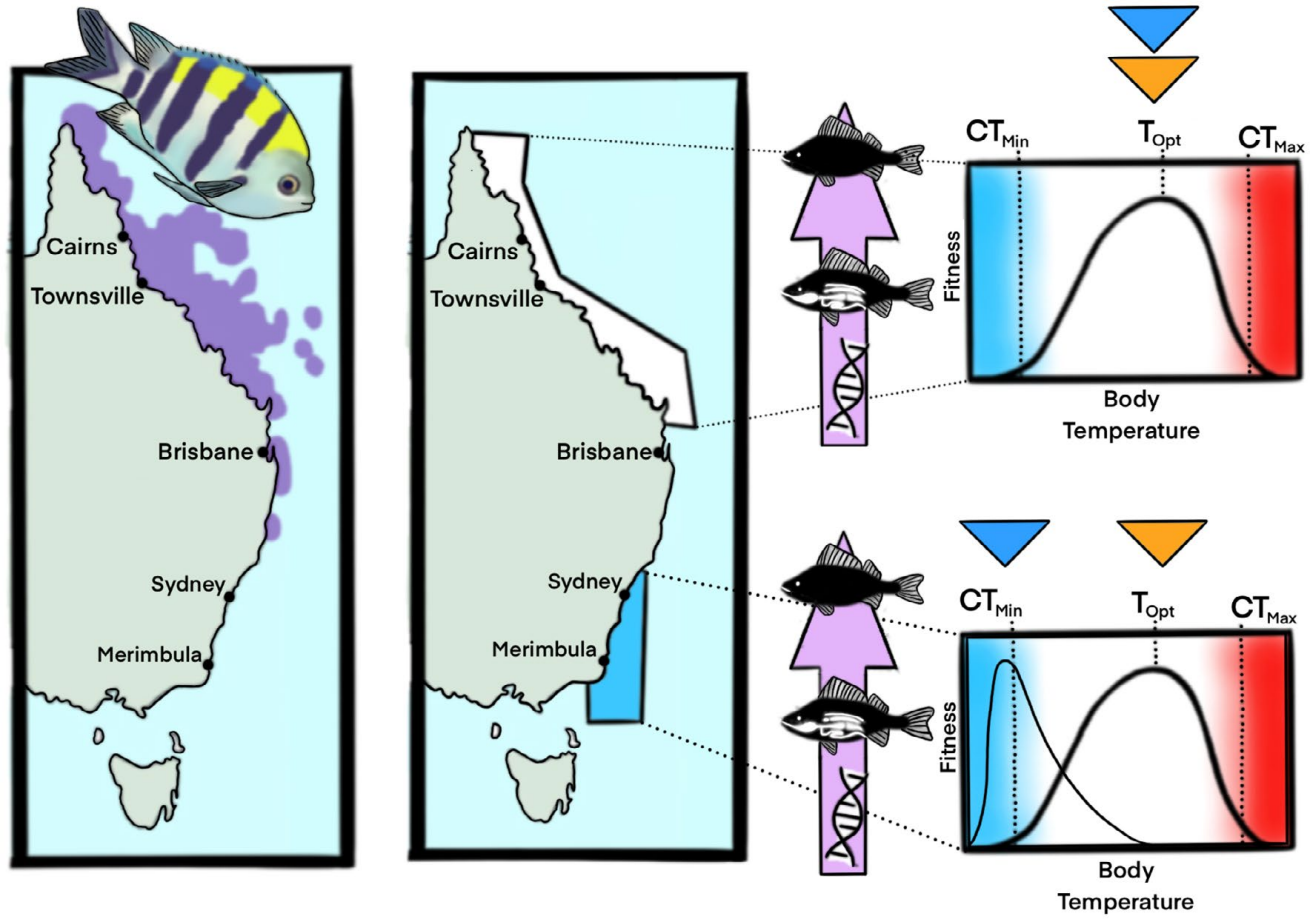
Habitat range of a coastal marine fish (Sergeant Major; *Abudefduf vaigiensis* ) under seasonal conditions prior to ocean warming events along the East coast of Australia. Habitat ranges (purple area on left panel) of northern Queensland down to northern New South Wales are within the optimal thermal tolerance limits (*T*_opt_) of the species during winter (blue triangle) and summer (orange triangle) as per the thermal performance curve, which is dictated by a cascade of physiological traits from cell to whole animal. Poleward movements down to southern New South Wales would hypothesize to be limited by the thermal tolerances of the species/populations. Summer conditions in southern New South Wales are within the species' thermal tolerances, but winter conditions are likely pushing the lower thermal limits (CT_min_), which would have sub‐lethal and lethal consequences on species function under these conditions. Map lines delineate study areas and do not necessarily depict accepted national boundaries. Current habitat range for *A. vaigiensis* inhabiting Australian East coast was adapted from FishBase (Froese and Pauly 2025; sourced from Aquamaps 2019, Kaschner et al. 2019). Fish and figure drawn using Procreate (Ver. 5.3.15) by ATD.

The persistence of a select few tropical species in temperate reefs (i.e., perhaps populations inhabiting the leading edge range) beyond their first winter predictably suggests that their physiology may be plastic enough across life‐history stages (i.e., larvae to adult) to tolerate cooler environmental temperatures (Figueira and Booth 2010; Figures 1 and 2). In contrast, populations found in warmer parts of their habitat range (e.g., northern GBR closer to equator; core and trailing edge populations) may not have such capacity (Donelson et al. 2019; Schmidt and Donelson 2024; Figure 1). For example, minimum sea surface temperature limits the poleward movements of tropical anemonefish populations, such as the Barrier Reef anemonefish ( *Amphiprion akindynos* ), but does not limit the movement of the more broadly distributed sub‐tropical species, the Wide‐Banded anemonefish ( *A. latezonatus* ) (Pryor et al. 2022) (Figure 2). Furthermore, most of the species that can overwinter in southern New South Wales (i.e., Merimbula) are sub‐tropical species (e.g., Yellow‐Back Puller *Chromis nitida* , and Moon Wrasse *Thalassoma lunare* ; Booth et al. 2018). Therefore, thermal tolerance may be plastic among populations of some tropical reef fish species, and there may be latitudinal differences in the physiological capacity for tropical fishes to adapt to cooler waters (Donelson et al. 2019; Schmidt and Donelson 2024) (Figures 1 and 2). While this may explain latitudinal population differences in thermal tolerances, this doesn't entirely explain why certain species can tolerate colder temperatures while others cannot if they originated from similar latitudes (Figure 3). Some tropical species can overwinter as far south as Sydney (e.g., Bluespine Unicornfish *Naso unicornis* ), whereas several species cannot (e.g., Yellow Surgeonfish *Acanthurus olivaceus* , Speckled Butterflyfish *Chaetodon citrinellus, and* Speckled damselfish *Pomacentrus bankanensis* ), despite having overlapping habitat ranges (Booth et al. 2018).

## 4 Thermal Stress on Range‐Shifting Tropical Fishes

**FIGURE 4:**
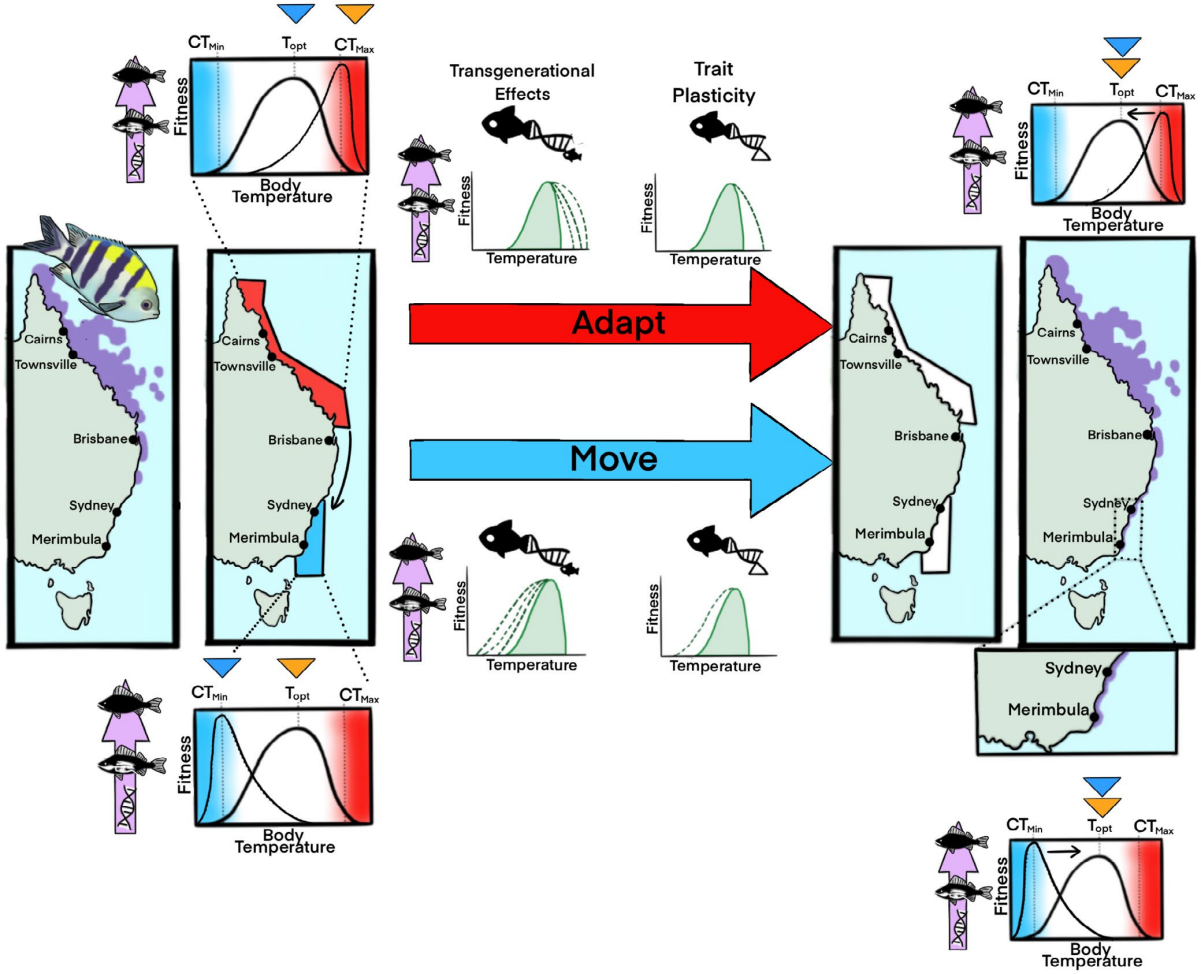
Habitat range of a coastal marine fish (Sergeant Major; *Abudefduf vaigiensis* ) under warming summer conditions along the East coast of Australia. Natural habitat ranges (purple area on left panel) of *A. vaigensis* extend from northern Queensland down to northern New South Wales. While winter conditions (blue triangle) are within the thermal optimum of the species, under summer (orange triangle) warming conditions, the upper thermal limits (CT_max_) of the species results in sub‐lethal and lethal consequences. Summer conditions in southern waters along the east coast of New South Wales are within the optimum thermal limits, but winter temperatures are within lower thermal limits (CT_min_) and would result in sub‐lethal and lethal consequences. Therefore, populations must either adapt to summer conditions, move polewards south, or succumb to warming and die if their physiology is not plastic enough for the prior options. Both adapting to warming summer conditions and movement poleward to southern winter conditions requires either physiological trait plasticity (cellular to whole‐animal traits are flexible in responding to thermal stress), transgenerational effects (thermal tolerance is passed onto future offspring) or both mechanisms. Physiological adaptations result in populations who have shifted their physiological tolerance limits back within optimum ranges under new habitat conditions. Under current warming conditions, *A. vaigiensis* populations have both remained in tropical habitats and successfully extended poleward into southern New South Wales by successfully overwintering in Sydney and Merimbula. Map lines delineate study areas and do not necessarily depict accepted national boundaries. Current habitat range for *A. vaigiensis* inhabiting Australian East coast was adapted from FishBase (Froese and Pauly 2025; sourced from Aquamaps 2019, Kaschner et al. 2019), and extended range into Sydney and Merimbula was adapted from RedMap Australia (RedMap Australia, https://www.redmap.org.au/species/1/3/, date of access: Sept 2024, Pecl et al. 2019). Fish and figure drawn using Procreate (Ver. 5.3.15) by ATD.

Tropicalization is a compelling physiological phenomenon because its mechanistic understanding requires a holistic view of an organism's entire thermal breadth. While warming pushes the upper thermal limits and initiates poleward redistributions, the “seeking refuge” component of tropicalization pushes the redistributing species' lower thermal limits (Figures 2, 3, 4). Critically, winter conditions may represent a population bottleneck for migrating species (Booth et al. 2018; Feary et al. 2014; Figueira and Booth 2010). We have established thus far that populations of sub‐tropical and tropical species inhabiting along the southern ranges of their habitat range are moving south at an alarming rate into Australia's temperate habitats; however, only a small number of species are successful at adapting long‐term to these conditions and surviving the first winter (see Booth et al. 2018). This next section will describe the thermal tolerances of tropical reef fish species from both ends of their thermal tolerance range. Importantly, we review the current state of knowledge on the physiological responses of vagrant species to temperate waters.

### 4.1 Impacts of Ocean Warming on Coral Reef Fish Physiology

Understanding physiological effects of ocean warming on tropical coral reef fish tolerance limits provides valuable context as to why tropicalization is occurring among populations of reef fishes inhabiting eastern Australia to begin with. The physiological, cellular, molecular, and transcriptomic impacts of climate warming pushing tropical fish's thermal maxima are well‐documented. Impacts of ocean warming (+1°C–3°C) on reef fish physiology include—but are not limited to—alterations in aerobic and anaerobic cellular pathways (Bernal et al. 2020; Johansen et al. 2021), an increase in basal metabolic demands (Johansen et al. 2024), reductions in aerobic scope (Johansen and Jones 2011; Nilsson et al. 2009; Rummer et al. 2014), reductions in body mass (Habary et al. 2017), reductions in swimming performance (Johansen and Jones 2011; Johansen et al. 2014), increases in spleen hemoglobin levels (Johansen et al. 2021), changes in gill structure (Johansen et al. 2021), reductions in hypoxia tolerance (Nilsson et al. 2010), and upregulation of heat‐shock proteins (Moore et al. 2023; Veilleux et al. 2018; Figure 2). Many cellular mechanisms are in place to mitigate and repair heat damage in fishes (e.g., heat‐shock proteins), and adaptation may be possible due to phenotypic plasticity (e.g., shift in heat tolerance to maintain swimming performance and aerobic scope; Johansen et al. 2024) and transgenerational acquisition of favorable traits and tolerances (Donelson et al. 2012; Fox et al. 2019) (Figure 2). However, migration of many tropical species poleward from the GBR occurs during elevated summertime temperatures (+3°C) combined with more intense and frequent heatwaves, which are pushing species' physiology beyond tolerable limits for many reef fish species in their natal habitat.

An important aspect of habitat range shifts is not solely poleward movements, but also vertical movements in the water column (Dahms and Killen 2023; MacDonald et al. 2016, 2018; Pinsky et al. 2013). The “deep reef refuge hypothesis” (DRRH) puts forth that species will seek deeper depths for refuge in response to warming shallow/surface temperatures (Calderón Aguilera 2023; Crosbie et al. 2019). Much of these observations have been taken from historical records of species abundance, presence/absence visual surveys and research trawls (Dahms and Killen 2023; Fredston‐Hermann et al. 2020), and primarily from temperate habitats (Caves and Johnsen 2021; Pinsky et al. 2020). In some temperate marine systems fishes are moving 1 m year^−1^ to deeper depths (Nye et al. 2009). On tropical reefs, the DRRH has received mixed support. Some studies have found support for the DRRH in tropical reef systems with many species shifting depths from shallow reefs to depths of 25–250 m in response to marine heatwaves and fishing pressures (e.g., Crosbie et al. 2019; Dahms and Killen 2023; Giraldo‐Ospina et al. 2020; Knott et al. 2025; Lenanton et al. 2017; Lindfield et al. 2016; MacDonald et al. 2016, 2018; Paxton et al. 2019; Pereira et al. 2018). Notable trade‐offs with selecting deeper depths versus shifting habitats polewards may include changes in sensory environment and cues (Caves and Johnsen 2021), increased bathymetric pressure (Smith and Brown 2002), and altered resource availability (Smith and Brown 2002). In contrast, butterflyfishes that selected deeper depths did not experience changes in physiological condition or increased territoriality, indicating that some species can successfully shift to deeper depths with minimal trade‐offs (MacDonald et al. 2018). However, other studies find no such support for DRRH for tropical species. Generally, some herbivore (Cure et al. 2021; Paxton et al. 2019), coral‐obligated (Jankowski et al. 2015; but see MacDonald et al. 2018, 2016) and reef symbiote (e.g., anemonefishes; Haguenauer et al. 2021) functional groups are restricted to shallow depths and do not appear to respond to stressors by distributing to deeper depths. Therefore, species restricted to shallow depths may need to have more plastic physiological responses to warming to remain in natural habitat ranges or else they must shift poleward to seek refuge or succumb to warming. These findings pose interesting hypotheses to test whether a pattern exists in which functional groups are shifting ranges poleward, and whether different reef fish functional groups have different tolerances to environmental stressors (e.g., see Miller et al. 2023).

### 4.2 Current State‐of‐Knowledge of Physiological Response of Tropical Vagrants to Temperate Conditions

Cold stress is rarely considered in physiological experiments for reef fish species inhabiting the GBR due to the relatively stable, warm seasonal habitats they thrive in. For species moving south, while temperate summer temperatures are within the natural thermal tolerance limits of tropical species (22°C–25°C), winter temperatures are approaching the critical thermal minima of most tropical species (< 20°C) (Eme and Bennett 2009) (Figures 2 and 4). In brief, under conditions pushing a fish's thermal minimum, the rate of biochemical reactions decreases, with many of the same systems impacted by heat stress affected, resulting in significant reductions in oxygen uptake rates, enzyme activity and protein synthesis, ion pumps and channel activities, diffusion rate of oxygen and metabolites, neural pathway signaling, swimming speed, digestion and cardiac output (Downie and Kieffer 2016; Downie et al. 2018; Suski and Ridgway 2009; Figure 2). The general strategy to acclimate to cold temperatures is to biochemically increase cell membrane fluidity (Reid et al. 2022) and increase enzyme and ATP production to compensate for energy production decline to support critical metabolic processes (e.g., osmoregulation and protein biosynthesis) and performance, primarily through mitochondrial biogenesis (Guderley 2004; Pörtner 2002). However, the biogenesis of ATP‐creating mitochondria in response to cold stress may increase risk of oxidative stress (O'Brien 2011). Indeed, cold stress is characterized by significant oxidative damage to cells due to the accumulation of reactive oxygen species (ROS) as a consequence of the increased oxygen solubility in cold temperatures (acute stress), decreases in mitochondria membrane fluidity disrupting electron transfer (acute stress), and the response to maintain mitochondria membrane fluidity through increases in polyunsaturated lipids concentration in membranes which are prone to increased oxidation (chronic stress; Kammer et al. 2011). Fish have developed several physiological responses to tolerate the build‐up of ROS and mitigate antioxidant damage, such as cellular production of antioxidants (Hoseinifar et al. 2020). Taken together, the foundations of understanding cold tolerance in fishes have unsurprisingly been studied in temperate and polar fishes that naturally live under these conditions. However, recent studies have adopted these responses to develop useful physiological, molecular, and transcriptomic biomarkers of cold stress for tropical species within an aquaculture setting (e.g., Chang et al. 2020; Liu et al. 2020, 2022, 2023; Lu et al. 2019; Wen et al. 2017). The response of vagrant reef fishes to overwintering conditions indeed is a critical bottleneck, but the underlying physiology is important for understanding the long‐term persistence of these species in temperate habitats.

Generally, studies have focused on investigating the overwintering physiology of tropical fishes on a small number of species, life stages, and traits. The most common species investigated is the sergeant‐major Damselfish (*A. vaigiensis*), which is among the few tropical species that have successfully overwintered in Sydney and as far south as Merimbula in New South Wales (Booth et al. 2018). However, other members of Pomacentridae and Chaetodontidae have also been studied. Laboratory and field experiments/in situ observations typically focus on comparing juvenile performance under temperate summer conditions (22°C–25°C; i.e., optimum thermal conditions) and winter conditions (18°C; beyond pejus temperatures; temperatures slightly beyond optimal tolerance limits where physiological compromises occur to maintain animal health and function; Fangue et al. 2021) measuring generally whole‐animal traits associated with energetics (oxygen uptake, growth, body condition), locomotion (predator escape response), and foraging (bite rate, diet selection, boldness). Winter conditions approaching thermal minima generally result in decreased feeding rates, decreased growth, increased cellular damage, and decreased swimming performance of juvenile vagrants (Djurichkovic et al. 2019; Figueira et al. 2009; Hayes et al. 2024; Kingsbury et al. 2020; Leriorato et al. 2021; Wolfe et al. 2020). Many studies have concluded that overwintering mortality in situ is most likely attributed to predation (smaller sizes, depressed escape responses and increased foraging to compensate for suppressed metabolism) and starvation/depletion of energy reserves associated with aforementioned physiological depression (Figueira et al. 2009, 2019; Leriorato et al. 2021), with a few studies proposing the possibility of mortality also associated with depressed metabolic rates (e.g., hypothermia). However, despite these lethal and sub‐lethal impairments, what mechanisms permit such survival for successful overwintering reef fishes?

Early evidence suggests that physiological trade‐offs and broadening diet selection may permit the survival of overwintering for some tropical fishes and may help buffer the physiological impairments associated with cold stress. For example, across a 750‐km gradient down the east coast of Australia (30.5°–37° S) there were no significant differences in body condition (Fulton's K, C:N ratios, protein content), but there was a decrease in performance (bite rate, activity levels, growth rate) across tropical species, with no such differences observed for temperate species along the same gradient (Kingsbury et al. 2020). Kingsbury et al. (2020) suggest that a trade‐off may exist whereby vagrant fish survive winter by prioritizing the maintenance of body condition over growth rate and activity, with normal growth rates resuming in summer under more optimal temperature conditions. Diet selectivity has been another mechanism suggested to enable vagrant fishes to survive winter. *A. vaigiensis* has been found to broaden its diet, habitat preference, and social behavior under temperate conditions, which may provide an advantage over other vagrant fishes (Coni et al. 2021; Kingsbury et al. 2020; Monaco et al. 2020). Specifically, under lower temperatures, juvenile *A. vaigiensis* selected a high‐protein diet that enabled them to decrease feeding activity while maintaining metabolic rate (Rowe et al. 2018). Herbivores, such as Convict surgeonfish ( *Acanthurus triostegus* ) buffer the effects of low temperature by selecting plant material high in nitrogen, which is important for growth (Miranda et al. 2019). Similarly, rabbitfish ( *Siganus fuscescens* ) from warm parts of Western Australia have flexibility in their gut microbiome, which helps colonize new colder environments and acquire novel food items beyond their existing habitat range (Jones et al. 2018). Decreases in activity and feeding and selection of food items of tropical fishes in cold waters suggest they have a behavioral strategy to maintain condition and reserves and may contribute to the success of some species shifting habitats. Vagrant fish in good body condition (i.e., adequate energy reserves) and faster growth rates may enjoy lower predation pressure and exploit dietary resources associated with being successful in novel temperate habitats (Miranda et al. 2019).

While the focus of this review is physiological responses of vagrant tropical reef species to temperate conditions, it is also important to highlight the changes in ecological processes that occur in temperate habitats in response to vagrants. Indeed, increased competition for resources (Miller et al. 2023), predator–prey interactions (Coni et al. 2022; McCosker et al. 2022) and habitat availability (Vergés et al. 2019; Vergés, Steinberg, et al. 2014; Barrientos et al. 2021) may have physiological ramifications on both local species and tropical vagrants. For example, tropical vagrant fishes exhibit more risk‐adverse behaviors, such as increased sheltering and flight initiation distances, in novel temperate reefs (Coni et al. 2022). Additionally, new tropical recruits tend to avoid kelp and macro‐algae as they may hide ambush predators (McCosker et al. 2022), and the continual removal of kelp due to increased herbivory from vagrants may promote increased juvenile settlement patches in temperate reefs (McCosker et al. 2022, Barrientos et al. 2021). Flexibility in schooling and behavior responses to conspecifics and local species, dietary generalism, or ability to exploit a range of micro‐habitats have been shown to be viable strategies for tropical vagrants to rapidly adapt to temperate habitats and outcompete local temperate species for resources (O’Connell et al. 2022, Monaco et al. 2020, Miller et al. 2023, Coni et al. 2021). Such adaptations may lower stress responses, such as plasma cortisol levels (see Section 5, “Primary Stress Response Biomarkers” below for details) for tropical vagrants due to adaptation to novel habitats, and increase stress responses of local temperate species due to increased competition for space and resources. Furthermore, removal of kelp due to significant increases in herbivory recently experienced on temperate reefs changes energy and nutrient cycling dynamics (Smith et al. 2021, Zarco‐Perello et al. 2017, 2019). For example, increased herbivory results in detritus build‐up (Zarco‐Perello et al. 2019) which may result in increased microbial activity leading to localized hypoxia (i.e., low‐oxygen), limiting oxygen supply for fish tissues (Attard et al. 2023). While the ramifications of these notable ecological phase shifts from kelp to barren reefs have yet to be fully realized and are an area of current interest, their cascading effects on the physiology of fish communities should be an area of additional research interest.

While fish that employ generalist strategies in behavior, diet, and trade‐offs in energy metabolism provide compelling evidence of the success of tropical vagrants in temperate habitats, cold temperature stress is a limiting factor despite the availability of resources that are abundant in temperate ecosystems (Leriorato et al. 2021). The critical thermal minimum of many tropical damselfishes is approximately 15°C (Eme and Bennett 2009), yet the overwintering threshold is several degrees warmer at 17°C–17.5°C, with most fishes unable to successfully overwinter (Booth et al. 2018; Figueira et al. 2009). Indeed, there is a discrepancy between minimum physiological tolerances measured in lab and field observations of species that exhibit successful vagrancy, as it would appear that species should not be successful under such depressed physiological states even if food is abundant (Figueira et al. 2009). However, most measured traits described above are whole‐animal physiological traits. There is a large knowledge gap involving the physiological responses at the cellular level of successful tropical vagrants to cold acclimation and how these systems fail in reef fishes that cannot overwinter. Investigating such cellular mechanisms is critical for several reasons. Firstly, whole‐animal function is governed by lower levels of biological organization, largely driven by genes that build proteins and enzymes that regulate cellular and molecular processes (Figures 2, 3, 4). For example, cellular damage due to cold stress will divert energy towards cell defense and repair and away from whole‐animal growth and body maintenance, which may have bottom‐up consequences on both individual and population fitness (Hayes et al. 2024). Secondly, changes in diet selection suggest that metabolic pathways for energy assimilation and production may be plastic in response to changing conditions for successful vagrants and suggest that unsuccessful vagrants may not have such metabolic pathway plasticity (Figure 4). Thirdly, investigating how aerobic and anaerobic metabolic pathways respond to cold stress may help develop biomarkers to help predict which species will be successful in migrating to colder waters, which have significant fishery and ecosystem management ramifications (Figures 2 and 4).

## 5 Physiological Framework for Understanding Range Shifts of Tropical Fish Species

The use of whole‐animal physiological traits has set an important foundation upon which to understand the mechanisms by which cold tolerance exists among some vagrant reef fishes. Moving forward, it will be critical to build upon these foundations by incorporating a range of traits across biological organization (i.e., cell to tissue to whole animal to population) through the incorporation of recent advancements in molecular, transcriptomic, and genetic techniques into our current toolbox of physiological techniques within ecological monitoring practices. Here, we develop a physiological framework to help guide future research in understanding the mechanisms underpinning the success of vagrants to overwinter in temperate habitats to improve predictive power for understanding poleward range expansions (e.g., Table 1).

**TABLE 1:** Examples of key physiological traits and their role in supporting a further understanding of climate‐driven redistribution of tropical fishes. Details include: Trait functions, example of metrics for quantifying traits, applicability of traits in experimental design, as well as the synergy of co‐measuring with other physiological traits at different levels of biological organization (ELS = early life stage; larvae and juveniles).

Trait category	Stress response category	Trait function	Example of traits/Biomarkers	Experimental design	Trait synergy	References
Hormonal stress response	Primary	Measure of stress response to environmental stimuli. Elevated epinephrine activates “fight or flight” responses. Cortisol activates long‐term stress response and can divert energy away from other key processes (growth, immune function)	Cortisol, epinephrine, norepinephrine	*Source:* Blood (adult) Whole body (ELS) *Design:* Compare between local and vagrant species (field) Cold‐Tolerance experiments (lab) *Life Stages*: ELS: newly arriving individuals (1st winter) Adult: Established vagrants vs. local temperate population (single measurement or repeated via tagging)	*Secondary* Oxidative stress/ROS concentration Lipid synthesis Glutathione Organ function Cellular cold adaption *Tertiary* Body growth Swimming performance Condition Metabolic rate Behavioural response *Ecological* Field samples Schooling behaviour Social interactions Predator‐prey interactions	Sadoul and Geffroy (2019)
Oxidative stress/Reactive oxygen species (ROS)	Secondary	Measure of cellular damage as a result of cold temperatures/cell response to increase membrane fluidity (e.g., increase long‐chain fatty acid synthesis)	ROS, DNA damage, lipid peroxidation (LPO) activity, Alanine Aminotransferase, cell apoptosis gene expression (e.g., p53)	*Source*: Blood (adult) Kidney (adult) Liver (adult) Muscle (adult) Skin (adult) Whole body (ELS) *Design*: Compare between local and vagrant species (field samples) Cold‐Tolerance experiments (lab), Dietary selection (lab/field) *Life stages*: ELS: newly arriving individuals (1st winter) Adult: Established vagrants vs. local temperate population (single measurement or repeated via tagging)	*Primary* Cortisol *Secondary* Antioxidants Energy storage pathways Energy metabolism Cell regulation/apoptosis Organ function Cellular function Cellular cold adapation *Tertiary* Body growth Condition Swimming performance Reproductive output *Ecological* Field samples	Cheng et al. (2017), Liu et al. (2022, 2023), Wang et al. (2022)
Antioxidants	Secondary	Measure of molecules that prevent oxidative stress to cells as a result of decreased water temperatures	Superoxide Dismutase (SOD), Catalase (CAT) *gene or metabolite	*Source*: Blood (adult) Muscle (adult) Gills (adult) Liver (adult) Whole body (ELS) *Design*: Compare between local and vagrant species (field) Cold‐Tolerance experiments (lab) Dietary selection (lab/field) *Life Stages*: ELS: are antioxidants produced? Adult: established vagrants versus local temperate population (single measurement or repeated via tagging)	*Primary* Cortisol *Secondary* Oxidative stress/ROS concentration Lipid/protein synthesis Glutathione Organ function Cellular cold adaption *Tertiary* Body growth Swimming performance Condition Oxygen uptake Reproductive output *Ecological* Field samples Dietary selection	Cheng et al. (2017), Liu et al. (2022), Gracey et al. (2004)
Metabolic pathway changes/Alternative energy storages	Secondary	Use of alternate energy sources/process metabolites differently under environmental change	Lactate dehydrogenase (LDH; use of anaerobic metabolism; gene and metabolite), mitochondria volume (citrate synthase), genes SLC25A5, SLC25A6, and SLC25A3 (mitochondria biosynthesis), genes CPT‐1, SCD‐1, FABP10 (fatty acid oxidation), cellular/mitochondrial respiration	*Source*: Muscle (adult) Kidney (adult) Liver (adult) Brain (adult) Gill (adult) Whole body (ELS) *Design:* Compare between local and vagrant species (field samples) Cold‐Tolerance experiments (lab), Dietary selection (lab/field) *Life Stages:* ELS: are larvae/juveniles capable of switching metabolic pathways or using alternative energy stores? Adults: Compare between local and vagrant species (field), Cold‐Tolerance experiments (lab), Dietary selection (lab/field)	*Primary* Cortisol *Secondary* Lipid/protein synthesis Carbohydrate synthesis Cellular cold adaption *Tertiary* Body growth Condition Swimming Reproductive output *Ecological*	Cheng et al. (2017), Wen et al. (2017), Sun et al. (2019), Liu et al. (2023), Hu et al. (2014), Chang et al. (2020), Gracey et al. (2004), Schnell and Seebacher (2008)
Protein function	Secondary	Protein/enzyme biosynthesis, function and degradation	Genes crbpalb, HSP70, rbm8a, prpf31, hmgb3a	*Source:* Muscle (adult) Kidney (adult) Liver (adult) Brain (adult) Gill (adult) Whole body (ELS) *Design:* Compare between local and vagrant species (field samples) Cold‐Tolerance experiments (lab), Dietary selection (lab/field) *Life Stages:* ELS: are larvae/juveniles capable of switching metabolic pathways or using alternative energy stores? Adults: Compare between local and vagrant species (field), Cold‐Tolerance experiments (lab), Dietary selection (lab/field)	*Primary* Cortisol *Secondary* Lipid/protein synthesis Carbohydrate synthesis Cellular cold adaption *Tertiary* Body growth Condition Swimming performance Reproductive output *Ecological* Field samples	Melis et al. (2017), Gracey et al. (2004), Cheng et al. (2017), Kyprianou et al. (2010), Liu et al. (2020, 2023)
Cellular cold adaptation	Secondary	Cellular response to increase tolerance to cold (e.g., increase membrane fluidity, protein function)	Genes HSP90 (protein response to cold stress), APO‐AIV, APO D, FABP (lipid transport), CYP51, GPAT (lipid biosynthesis)	*Source*: Muscle (adult) Kidney (adult) Liver (adult) Brain (adult) Gill (adult) Whole body (ELS) *Design:* Compare between local and vagrant species (field samples) Cold‐Tolerance experiments (lab), Dietary selection (lab/field) *Life Stages* ELS: are larvae/juveniles capable of switching metabolic pathways or using alternative energy stores? Adults: Compare between local and vagrant species (field), Cold‐Tolerance experiments (lab), Dietary selection (lab/field)	*Primary* Cortisol *Secondary* Lipid/protein synthesis Cellular oxygen uptake Antioxidant production Oxidative stress/ROS concentration DNA damage glutathione *Tertiary* Body growth Condition Swimming performance Reproductive output *Ecological* Field samples ield samples	Hu et al. (2014), Liu et al. (2022, 2023), Sun et al. (2019), Gracey et al. (2004)
Organ function	Secondary	Maintenance and health of tissues	Alanine transaminase (ALT), Aspartate transferase (AST) *gene or metabolite, gene CIRBP	*Design*: Compare between local and vagrant species (field) Cold‐Tolerance experiments (lab) *Life Stages*: ELS: newly arriving individuals (1st winter) Adult: Established vagrants vs. local temperate population (single measurement or repeated via tagging)	*Primary* Cortisol *Secondary* Lipid/protein synthesis Energy metabolism pathways Cellular cold adaption *Tertiary* Growth Body condition Swimming performance Reproductive output *Ecological* Field samples	Wen et al. (2017), Cheng et al. (2017), Sun et al. (2019), Gracey et al. (2004)
Body growth, condition	Tertiary	Contributes to increasing size, overall individual health	Mass, length, Fulton's K	*Source*: Whole body (adult & ELS) *Design*: Compare between local and vagrant species (field) Cold‐Tolerance experiments (lab), Dietary selection (lab/field) *Life Stages*: ELS: body condition (newly arrived vs. 1st winter in field, cold tolerance in lab), Growth rates (cold tolerance in lab) Adult: body condition/size/growth rate (tagging in field over time, cold tolerance experiments in lab)	*Primary* Cortisol *Secondary* Oxidative stress/ROS concentration Lipid/protein synthesis Energy storage metabolic pathways Energy metabolism pathways Cellular oxygen uptake Organ function Cellular cold adaption *Tertiary* Swimming performance Reproductive output *Ecological* Field samples Dietary selection	Figueira et al. (2009), Djurichkovic et al. (2019), Kingsbury et al. (2020)
Swimming performance	Tertiary	Locomotion associated with prey capture, predator avoidance, migration and daily movement patterns	Burst swimming/escape response (predator avoidance), critical swimming speed (U_crit_; universal measure of upper swimming speed for comparative purposes), endurance (long‐term swimming), routine swimming (unprovoked swimming)	*Source*: Whole body (adult & ELS) *Design*: Impacts of winter temperatures on swimming performance *Life Stages*: ELS: Burst, endurance, U_crit_ for 1st winter Adult: Ucrit, endurance of established vagrants (lab), routine swimming (underwater cameras; field)	*Primary* Cortisol *Secondary* Oxidative stress/ROS concentration Organ function Lipid/protein synthesis Energy storage metabolic pathways Energy metabolism pathways Cellular oxygen uptake Cellular cold adaption *Tertiary* Oxygen uptake Body growth Condition Reproductive output *Ecological* Field observations	Downie and Kieffer (2017), Downie et al. (2020), Illing et al. (2021), Figueira et al. (2009), Djurichkovic et al. (2019), Leriorato et al. (2021)
Oxygen uptake	Tertiary	Delivery of oxygen to tissues; proxy for energy expansion	Standard metabolic rate (SMR; resting oxygen uptake), Routine Metabolic Rate (RMR; oxygen uptake during normal activity), Maximum Metabolic Rate (MMR; oxygen uptake during activity)	*Source*: Whole body (adult and ELS) *Design*: Impacts of winter temperatures on oxygen uptake *Life Stages*: ELS: oxygen uptake during 1st winter Adult: compare energy demands of vagrant and established fishes (field and lab respirometers)	*Primary* Cortisol *Secondary* Oxidative stress/ROS concentration Organ function Lipid/protein synthesis Energy stroage metabolic pathways Energy metabolism pathways Cellular oxygen uptake Cellular cold adaptation organ *Tertiary*	Rowe et al. (2018), Downie, Phelps, et al. (2021), Downie, Lefevre, et al. (2023), Downie, Phelps, et al. (2024)
Reproductive output (RO)	Tertiary	Amount of reproductive material (sperm or eggs) produced	Gonadosomatic index (GSI), egg production, spawning biomass	*Source*: Whole body (adult only) *Design*: Measure egg production, GSI, spawning biomass from adults kept under winter temperatures (lab) Measure GSI, spawning biomass from field samples (single or repeated over time with tagging) *Life Stages*: Adult only (see “Design”)	*Primary* Cortisol *Secondary* Oxidative stress/ROS concentration Organ function Lipid/protein synthesis Energy storage metabolic pathways Energy metabolism pathways Cellular oxygen uptake Cellular cold adaptation *Tertiary* Body growth Condition Oxygen uptake Swimming performance *Ecological*	Thorsen et al. (2003)
Critical thermal limits	Tertiary	Determines upper and lower temperature tolerances	Critical thermal minima (CT_min_) and critical thermal maxima (CT_max_)	*Source*: Whole body (adult & ELS) *Design*: Impacts of temperate winter and summer temperatures on newly arriving vagrants Determine thermal minima of tropical and sub‐tropical species inhabiting leading, core and trailing edges to evalaute capacity to adapt to temperate conditions *Life Stages:* ELS and adult (lab)	*Primary* Cortisol *Secondary* Oxidative stress/ROS concentration Organ function Lipid/protein synthesis Energy storage metabolic pathways Energy metabolism pathways Cellular oxygen uptake cellular cold adaptation *Tertiary* Body growth Condition Reproductive output Swimming performance Oxygen uptake *Ecological* Field observations	Illing et al. (2020), Eme and Bennett (2009)

### 5.1 Step 1: Define Appropriate Study Aims

Understanding tropicalization can generally be divided into two broad aims: 1. Determine the long‐term persistence of currently observed tropical species in temperate habitats or 2. Determine whether leading edge populations of species who have not yet expanded their ranges polewards could survive in temperate regions. Both situations require proper management intervention to select model organisms (e.g., important fishery species moving south, ecological keystone species, invasive species etc.) to determine the overall aim. Experimental approaches to address both broad aims would involve either “cold‐tolerance experiments” under laboratory settings or field sampling. Cold‐tolerance experiments would involve exposing species of interest (tropical species currently found in temperate habitats or have yet to move but have the potential to) to winter temperatures (15°C–18°C), preferably over a long‐time span (i.e., few weeks to month(s)) to determine chronic stress response and potential to acclimatize to conditions. However, some cellular and molecular responses may be evident within a few hours or days of exposure (e.g., cortisol). Field sampling would involve taking samples (e.g., blood, gill clip or morphometrics) from animals caught in the field and would be a valuable approach for monitoring programs to assess, for example, how different physiological biomarkers change across season for newly arrived vagrants or established vagrants. A locally adapted temperate species should act as an experimental control/baseline for both lab and field experiments to compare how physiological traits differ between a vagrant species adapting to local conditions and the measurable expression of those traits in a species that has locally adapted. The selection of physiological biomarkers and the duration of experiments will ultimately be decided upon the study aims (e.g., survival, reproductive potential, changes in daily performance such as swimming and behavior; see Step 4 and Table 1).

### 5.2 Step 2: Identify Study Species and Use of Valuable Sentinel Species

Certain species have been consistently identified in visual surveys as prominent vagrants in tropical waters (e.g., *A. vaigensis*, rabbitfishes) and have been common study species in laboratory experiments (Booth et al. 2018; Djurichkovic et al. 2019; Figueira et al. 2009; Vergés, Tomas, et al. 2014). Commonly observed vagrants suggest their physiology is adaptable to colder conditions and should be considered for use in field and lab experiments. Additionally, temporal changes in these vagrant population numbers may suggest the extent to which their physiology is adaptable to colder conditions over longer periods. Therefore, species like *A. vaigensis* and other commonly identified tropical fishes could be considered a sentinel species for tropicalization, as based on current lab studies and field observations they respond to chronic cold stress in a predictable manner (Adams and Ham 2011) and can be used to monitor the extent by which physiological changes mediate long‐term persistence of tropical species in temperate conditions. Ecological monitoring techniques such as visual surveys/monitoring programs, historical data sets, and even use of eDNA to detect the presence of species difficult to visually observe (e.g., Downie, Bennett, et al. 2024) will be valuable in determining focal animals.

### 5.3 Life Stage Considerations

It is important to note that most of the aforementioned studies on metabolic pathway alterations in response to cold tolerance of tropical and sub‐tropical fishes have been performed on juvenile and adult fishes. Since larvae are the dispersive stages that travel from tropical to temperate habitats during the summer months, understanding the flexibility of their physiology over early development will be critical (Booth et al. 2018). Indeed, an important factor to consider in terms of energy metabolism especially is whether vagrant larval and early juvenile tropical fishes moving into temperate waters have the physiological capacity to shift their metabolic pathways or whether their metabolic pathways are fully developed to respond to stress, particularly cold stress. Indeed, many metabolic pathways change when reef fishes settle onto benthic reef habitats (Downie, Lefevre, et al. 2023; Downie, Phelps, et al. 2024; Huerlimann et al. 2024; Roux et al. 2023), and their physiology is quite sensitive to disturbance (e.g., Downie, Phelps, et al. 2021). For example, anaerobic glycolysis genes are poorly expressed during the beginning of the development of Malabar grouper ( *Epinephelus malabaricus* ) ontogeny but increase during development (Huerlimann et al. 2024). Young grouper larvae mainly rely on citric acid cycle for aerobic energy production and then switch progressively to anaerobic energy production via glycolysis and lactic fermentation during metamorphosis (Huerlimann et al. 2024). In contrast, clownfish ( *Amphiprion ocellaris* ) larvae use glycolysis and fermentation, and switch to fatty acid mediated β‐oxidation and citric cycle via thyroid hormones during metamorphosis which produces more energy for development (Roux et al. 2023). It is widely uncertain whether young reef fishes can switch between metabolic pathways and utilize alternative energy production outside of developmental functions in response to cold stress. For example, in response to heat stress, anemonefish ( *Amphiprion melanopus* ) and spiny chromis ( *Acanthochromis polyacanthus* ) larvae did not increase lactate dehydrogenase (LDH; catalyzes the interconversion of pyruvate and lactate to generate energy from anaerobic metabolism) or citrate synthase (a biomarker for mitochondria abundance and aerobic metabolism), which would typically increase in older life stages (Illing et al. 2020), possibly indicating that compensatory mechanisms to thermal stress aren't yet developed in early life stages of these specific tropical reef species or may not be ubiquitous among tropical species at this stage. Many reef species will likely be dispersing during summer, in which temperate water conditions are similar to tropical summer conditions. However, nocturnal temperatures, unexpected cold spikes, and the short transition to winter conditions may prove challenging for newly arriving tropical species’ early life stages. Experiments should consider a range of life histories when determining the persistence of tropical vagrants. Light traps are a common ecological methodology used on tropical reefs to capture newly arriving larvae to reefs (Downie, Phelps, et al. 2024; Downie, Phelps, et al. 2021; Meekan et al. 2001) and can be used to not only capture individuals for experiments but also monitor the influx of young fish to temperate ecosystems for monitoring and record purposes, which would be valuable for management. Taken together, younger life stages can be used to determine whether moving species have the innate mechanisms to survive winter conditions, and older life stages can be used to determine long‐term persistence.

### 5.4 Step 4: Physiological Biomarker Selection Across Multiple Levels of Biological Organization

The ability of an organism to function and respond to its environment is the product of a cascade of bottom‐up traits from cellular processes to tissue and organ systems to whole‐animal functions that have developed over evolutionary history. As previously described, cellular and molecular processes set the pace of metabolic processes which directly impact the rate at which organs and tissues operate, which in turn limits whole‐animal performance, development and function (Figure 2). For ectotherms, like fishes, these processes are directly related to environmental temperature (Figures 2, 3, 4). An animal's response to external stress is integrated with the same levels of biological organization and can be generally categorized as a primary (e.g., neuroendocrine responses such as stress hormones), secondary (changes at cellular/molecular and tissue/organ level, such as metabolism, blood chemistry, osmoregulation, immune system, cardiac), or tertiary (whole‐organism health and population changes) response (Figure 2) (Barton 2002). The current knowledge base for understanding physiological responses under tropicalization has primarily focused on tertiary responses such as growth, development, whole‐animal metabolic rate (i.e., energy intake through oxygen uptake) and swimming performance (Djurichkovic et al. 2019; Figueira et al. 2009; Hayes et al. 2024; Kingsbury et al. 2020). While these traits are very valuable and have advanced our understanding of tropicalization, moving forward, we need to incorporate lower levels of biological organization at the cellular and molecular level into experimental design, as changes at the cellular level have significant bottom‐up impacts on whole‐animal function and impacts fitness at the population level (Figure 2). This mechanistic approach incorporating “trait synergy” between different levels of biological organization can provide critical information as per species' long‐term adaptive capacity to cold temperatures (Table 1). A suite of valuable physiological measurements, from cell to whole animal, and the synergy between them can therefore be used as reliable biomarkers managers can use to monitor adaptive capacity of new vagrants to an area.

A point of consideration is the repeatability of measurements over time when designing experiments and selecting physiological traits. Many traits can be taken at a single point of time but are far more valuable when repeated measures on the same individual are taken temporally. This will be especially important for determining acclimation and adaptability of vagrants for monitoring purposes (e.g., do primary, secondary or tertiary stress responses change over time), and can reflect metabolic/transcriptomic changes in new habitats linked with critical life‐history processes such as reproduction, survival, and movement patterns. Many of the traits described in the subsequent section can be repeatedly sampled, non‐invasively, in wild‐caught fish. This includes blood samples to measure serum metabolites and body morphology. A valuable methodology is taking small clips from gill filaments to measure changes in the transcriptome (Jeffries et al. 2021). While brain, liver, and somatic tissues have historically been favorable tissues to sample and they do offer valuable insights into transcriptome changes in response to environmental stressors; however, they generally result in lethal endpoints. Gills are an ideal tissue as they are non‐invasive to sample (i.e., grow‐back) and are a multi‐faceted organ that is in direct contact with the external environment (Akbarzadeh et al. 2018; Jeffries et al. 2021). Partnered with telemetry or tracking technologies, numerous measurements on vagrant species could be made to document their adaptive capacity to temperate habitats.

#### 5.4.1 Primary Stress Response Biomarkers

Measurements of hormones, primarily serum cortisol, as well as catecholamines (epinephrine and norepinephrine) have been valuable biomarkers for evaluating stress associated with environmental changes in animals, including fishes (Harper and Wolf 2009; Lemos et al. 2023). Elevated cortisol and catecholamines indicate that energy usage is directed towards biochemically responding to a stressor, which is a normal physiological response in the short term. However, chronic stress diverts energy away from routine energy expenditure (e.g., growth, development, tissue/cellular repair and maintenance), which has severe consequences on individual health and function (Narayan 2017). Elevated cortisol—generally taken from blood—has been found in fishes exposed to cold temperatures (reviewed by Reid et al. 2022). In short, stress hormones are elevated during initial cold shock and may not return to pre‐shock levels if cold exposure is chronic (He et al. 2015; Ji et al. 2016; Reid et al. 2022), and therefore have long‐term consequences on individual fitness. Therefore, measuring stress hormones such as cortisol and catecholamines in newly arrived or established vagrants and comparing values to native temperate populations may provide an indication of whether a vagrant's metabolic pathways have fully adapted/acclimated to colder temperatures. Measuring primary stress responses synergistically with secondary or tertiary stress response(s) is highly preferred as it shows the cascading effect of elevated cortisol on other biochemical or physiological processes, which may either compromise a tropical species' ability to locally adapt to temperate conditions if levels remain elevated or, if levels decrease, may contribute to adaptation (Table 1). Cortisol, epinephrine, and norepinephrine can be easily measured non‐invasively via blood samples from lab or field samples.

#### 5.4.2 Secondary Stress Response Biomarkers

Recently, there has been a significant interest in understanding metabolic pathway alterations in response to cold tolerance of tropical and sub‐tropical aquaculture species from south‐east Asia and China, due to unpredictable cold snaps resulting in large‐scale mortalities and billions of dollars of stock lost (Chu et al. 2021; Liu et al. 2020, 2022, 2023). Many of these studies utilize rapidly advancing technologies such as metabolomics (study of changes in metabolites and associated pathways in cells, tissues and organ systems in response to cellular processes and environmental change) and transcriptomics (study of RNA which are copies of DNA which create proteins that have critical cellular functions) to help develop biomarkers based on changes in cellular and physiological pathways that can predict the success of species and individuals exposed to cold conditions (Cheng et al. 2017; Chu et al. 2021; Liu et al. 2020, 2022, 2023; Ripley et al. 2024; Wen et al. 2017). This has enabled aquaculture managers to better understand which species can be effectively kept as aquaculture species under such fluctuating and unpredictable conditions, and selectively breed cold‐tolerant individuals within a population for increased cold‐tolerant offspring (Kyprianou et al. 2010). While it is important to note that cellular and molecular responses to cold stress are highly species‐specific with respect to individual genes and metabolites impacted (i.e., no “one glove fits all” for all species), there are several common pathways altered across species. Specifically, pathways associated with energy metabolism (e.g., Gracey et al. 2004; Hu et al. 2014; Lu et al. 2019; Melis et al. 2017), lipid metabolism (energy usage and cell wall fluidity), and amino acid metabolism (e.g., Gracey et al. 2004) were among the most ubiquitous among multiple species for survival. It is important to note that other systems associated with cell signaling, cell apoptosis, tissue re‐modeling and immune response play critical roles in cold response, but for brevity, they are mentioned here but not described in detail further. The focus of this section on secondary stress responses is to introduce a range of measurable traits to a wider audience of their usefulness for understanding tropicalization. Furthermore, discussing the synergy between lower (primary and secondary responses) and higher (tertiary and community/population level) levels of biological organization will be critical for experimental design (Table 1).

##### 5.4.2.1 Biomarkers for Metabolic Pathway Changes

Tertiary stress measurements of growth, development, metabolic rate, and locomotion in response to cold adaptation should consider whether metabolic pathways that process and deliver energy are altered. Under cold stress, several species switch or utilize alternative energy storages and metabolic pathways to generate energy. For example, several species elevate the activity of anaerobic metabolic processes such as anaerobic glycolysis (break down of sugars to pyruvate in the absence of oxygen) to save energy reserves during stressful cold responses or increase/maintain energy production under decreased metabolic rates or stress (Wang et al. 2022; Wen et al. 2017). Since oxygen uptake decreases under low temperatures, the mismatch of oxygen supply and tissue demand results in the onset of anaerobic metabolism to support energy production under cold conditions (Chang et al. 2020; Song et al. 2019). Cold stress increases anaerobic biomarkers such as LDH activity in the brain, kidney, muscle, and liver, as found in pufferfish ( *Takifugu obscurus* ; Cheng et al. 2017), Discus fish (*Symphysodon aequifasciatus*; Wen et al. 2017), orange‐spotted grouper (*Epinephelus coicoides*; Sun et al. 2019), carp ( *Cyprinus carpio* ; Gracey et al. 2004), *Gymnocyprinus prezwalskii* (Liu et al. 2023), flounder ( *Paralichthys olivaceus* ; Hu et al. 2014), milkfish ( *Chanos chanos* ; Chang et al. 2020), and tilapia ( *Oreochromis mossambicus* ; Schnell and Seebacher 2008). However, anaerobic metabolism yields significantly lower amounts of energy than carbohydrate (i.e., aerobic) metabolism (Chang et al. 2020), and thus cold‐tolerant species likely use anaerobic metabolic pathways as supporting systems for energy production or trade‐off energy‐intensive processes to reduce energy (Gracey et al. 2004). However, some species elevate aerobic metabolism under cold stress. Carp, for example, increase mitochondria in their skeletal muscles under cold stress to maintain aerobic energy metabolism, as evident by increased expression of the genes SLC25A5, SLC25A6, and SLC25A3, which supply ADP and Pi to ATP synthase (Gracey et al. 2004).

##### 5.4.2.2 Biomarkers for Energy Storage

Complementing measurements of growth and development are whether fish use alternative energy stores to deliver energy under cold stress. Many tropical species under cold snaps generally use lipid (fat) stores and break down fatty acids via aerobically driven β‐oxidation in the mitochondria for usable energy (examples include Gilthead seabream *Sparus aurata* Kyprianou et al. 2010; tiger barb *Puntigrus tetrazona* Liu et al. 2020; longsnout catfish *Plicofollis argyropleuron* Liu et al. 2022; *G. prezwalskii* Liu et al. 2023; zebrafish *Danio rerio* Lu et al. 2019; *C. carpio* Gracey et al. 2004). Similarly, many tropical and sub‐tropical fish that utilize lipid stores for energy under cold stress also increase LDH activity, suggesting a complementary system to generate energy; examples include *S. aequifasciatus* (Wen et al. 2017), *E. coicoides* (Sun et al. 2019), *G. prezwalskii* (Liu et al. 2023), and *P. olivaceus* (Hu et al. 2014). Critical genes of interest include the expression of CPT‐1, SCD‐1, and FABP10, which are critical in ATP production from fatty acid oxidation (Sun et al. 2019). Some fish, like *O. mossambicus* , appear to rely more on lipid metabolism under cold stress, as evident by decreases in gene expression associated with the citric acid cycle or employ feeding cessation and rely on lipid catabolism and autotrophy to supplement energy for function and reduce cell damage (Lu et al. 2019). In contrast, yellow croaker (*Larimichthy crocea*) enhances carbohydrate metabolism under cold stress, which may be a viable strategy if enough oxygen and carbohydrates can enter the system (Qian and Xue 2016).

##### 5.4.2.3 Biomarkers for Adapting/Acclimating to Cold Conditions

Cold adaptation requires several cellular and biochemical changes which can be measured in terms of changes in cell architecture and stress response (Guderley 2004; Reid et al. 2022); which can complement both measurements of individual performance as well as support ecological surveys to predict the persistence of commonly observed vagrants (Table 1). Cold temperatures reduce the fluidity of membranes of cells, impacting cell signaling and diffusion of materials into and out of cells. While lipids are a source of energy as previously discussed, the biosynthesis of long‐chain unsaturated fatty acids maintains membrane fluidity under cold stress and is a critical mechanism that may permit some fishes—especially tropical and sub‐tropical species—to survive cold stress (Liu et al. 2022). Example species exhibiting an increased gene expression of lipid synthesis or measurement of lipid metabolites includes cold‐tolerant *P. argyropleuron* (Liu et al. 2022), *G. prezwalskii* (Liu et al. 2023), *E. coicoides* (Sun et al. 2019), *C. carpio* (Gracey et al. 2004), and *P. olivaceus* (Hu et al. 2014); with genes of interest including APO‐AIV, APO D, FABP (lipid transport), CYP51, GPAT (lipid biosynthesis; Hu et al. 2014). While these unsaturated fatty acids do maintain cellular membrane integrity, they are also prone to high oxidation and increase intercellular ROS production which causes oxidative stress to proteins, DNA, and fatty acids. To compensate, many species increase the production of antioxidants under cold temperature stress to counteract the effects of increased ROS production from unsaturated fatty acids in cell membranes, and the increased solubility of oxygen in cold water (Cheng et al. 2017; Liu et al. 2022, 2023; Wang et al. 2022). Many biomarkers of oxidative stress include increased serum antioxidant concentrations or gene expressions such as SOD and CAT (Cheng et al. 2017; Liu et al. 2022; Gracey et al. 2004), and metabolites such as glutathione (Liu et al. 2023; Sun et al. 2019). Alternatively, measuring DNA damage via comet assays can be used as valuable biomarkers to evaluate DNA damage from winter cold stress in native and vagrant species (Sun et al. 2019).

##### 5.4.2.4 Biomarkers for Protein Function

Given the wide variety of roles proteins play in maintaining organism function (e.g., enzymatic activity), understanding how protein function and integrity respond to cold stress will be critical in evaluating the success of an animal's function and health under cold stress. Several valuable amino acid metabolism biomarkers can be used to evaluate protein biosynthesis, folding, and degradation. Generally, under cold stress, increases in enzyme production maintain cellular performance, which requires energy, possibly from endogenous alternative stores such as lipids (Gracey et al. 2004). Species such as *G*. *prezwalskii* (Liu et al. 2023) and *O. mossambicus* (Yang et al. 2015) experience changes in gene expression associated with nucleic acid synthesis/metabolism (decrease in DNA replication, ribosome biogenesis, RNA degradation, RNA polymerase, RNA transport; increase in DNA repair) and amino acid metabolism and protein synthesis (increase in proteasome subunits, and ubiquitin synthesis‐related pathway) under cold stress to promote protein formation as compensation for decreased metabolic rates. However, under cold stress some species (e.g., seabream and carp) promote protein catabolism (especially for damaged proteins), and therefore the presence of high amounts of mobilized free amino acids may be used as a biomarker for the breakdown of proteins to be absorbed by tissues for storage or energy (Melis et al. 2017; Gracey et al. 2004). Additionally, genes of interest such as *crbpalb* (cold‐inducible RNA binding protein), HSP90 (indicated that proteins are impaired and ability of cells to respond to cold stress), HSP70 (aids in protein folding, regulating, secretion and degradation under cold stress), rbm8a (RNA binding motif protein 8A), prpf31 (PRP pre‐mRNA processing factor 31), and hmgb3a (high‐mobility group Box 3a) may be valuable biomarkers to evaluate defenses against cold shock with respect to protein biosynthesis and integrity (Cheng et al. 2017; Kyprianou et al. 2010; Liu et al. 2020, 2023).

##### 5.4.2.5 Biomarkers for Organ Function

Several serum biomarkers exist that can be used to determine whether cold stress impacts general organ function. Common amino acid biomarkers for stress include ALT (alanine transaminase) and AST (aspartate transaminase), which are amino acid transferases that are released into the plasma, indicating organ dysfunction and can also mobilize amino acids as alternative energy sources under stress (Wen et al. 2017; Cheng et al. 2017; Sun et al. 2019). Additionally, the presence or change of expression of cold‐shock proteins such as CIRBP (a universal indicator of cold exposure in vertebrates) is a valuable biomarker that is conserved across teleost species (Gracey et al. 2004).

#### 5.4.3 Tertiary Stress Responses Biomarkers

Tertiary stress responses are widely used and valuable metrics to evaluate vagrant responses to temperate conditions. Examples of measurements include somatic measurements of growth/condition and development (Figueira et al. 2009; Djurichkovic et al. 2019; Kingsbury et al. 2020), whole‐animal measures of oxygen uptake (proxy for metabolic rate; Rowe et al. 2018), swimming performance (Figueira et al. 2009; Djurichkovic et al. 2019; Leriorato et al. 2021) and have been discussed previously. Additional tertiary responses would include whole‐animal thermal limits, such as critical thermal methodologies (Eme and Bennett 2009; Illing et al. 2020), which test the upper and lower limits of a species, and can help predict species range extensions (Strader et al. 2023), yet have not been extensively tested on tropical species (but refer to Eme and Bennett 2009). The importance of these aforementioned measurements or their interpretation has been reviewed elsewhere in the physiology and fisheries literature. The value of measuring these tertiary traits within the context of tropicalization is the magnitude at which winter temperatures will limit whole‐animal function, and the consequences on population fitness if vagrants become established in temperate habitats. Furthermore, the value of pairing tertiary responses with primary and secondary responses in the laboratory and field will help provide the mechanistic explanation for the temperate persistence of tropical vagrants through the bottom‐up effect of cellular, genetic, and molecular adaptations that directly control whole‐animal performance (Table 1). Tertiary responses synergistically measured with primary and/or secondary responses have the great potential to create a robust sampling framework (i.e., described above and Table 1) for understanding how tropical vagrants adapt to temperate conditions that can be packaged to environmental monitoring programs, conservation efforts, and fisheries management, since tertiary measures (e.g., growth, reproductive output) are generally implemented into these programs, and many primary and secondary measures can be non‐invasively co‐measured.

### 5.5 Step 5: Considering Interactive Effects With Ecological Processes and Temperate Environmental Stressors

Mesocosm experiments involving novel social behavior between local and vagrant species have the benefits of measuring multiple physiological metrics interacting with behavior. As highlighted above in Section 4.2, ecological processes such as predator–prey interactions (McCosker et al. 2022), changes in social behavior (Coni et al. 2022) and competition for resources (Miller et al. 2023) should be considered as well and can complement many physiological metrics, such as primary stress responses (e.g., cortisol; Table 1). For example, novel interactions between pairing local temperate and vagrant tropical reef fishes have been shown to alter feeding rates, growth rates, activity, and oxidative damage versus isolated fishes, highlighting that behavioral and social dynamics between tropical and temperate species can directly influence fitness (Sasaki et al. 2024). Laboratory measures of social and behavioral interactions can be partnered with underwater videos on temperate reefs to highlight whether these confined interactions in the lab are occurring in nature.

Another important consideration is other abiotic environmental conditions besides temperature, which has been the focus of this review. Indeed, tropical, sub‐tropical, and temperate coastal habitats vary in terms of dissolved oxygen (Clarke et al. 2022; Fusi et al. 2024), pH (González‐Durán et al. 2021), visual and sensory cues (Cortesi et al. 2020), ultraviolet radiation (Downie, Wu, et al. 2023; Hird et al. 2024), and turbidity (Figueroa‐Pico et al. 2020), among others. Recent studies have emphasized that experimental and field‐based biological research should incorporate multiple, interactive stressors in research design (Alton and Franklin 2017; Ban et al. 2014; Downie, Cramp, et al. 2024; Hird et al. 2024). Therefore, exposing tropical and sub‐tropical species from leading edge populations to temperate conditions inclusive of a range of abiotic conditions will provide a more holistic view of how different interactive stressors will impact vagrant fishes ability to adapt to their new habitats. Additionally, these aforementioned stressors may contribute to the species‐specific success and failure to adapt to cold, temperate conditions.

### 5.6 Step 6: Integration of Physiological Biomarkers With Ecological Techniques and Monitoring: Management Perspective

Presence of tropical vagrant fishes in temperate waters has significant implications for marine management and policy (Bonebrake et al. 2018). With an enhanced understanding of physiological traits and how these scale with environmental conditions, resource managers can better anticipate and respond to the redistribution of marine species. One primary use of this information is to establish adaptive management strategies that account for both transient and permanent shifts in fish populations. For example, monitoring programs can be enhanced by incorporating predictions of vagrant species' movements, particularly during anomalous warming events such as marine heatwaves (Champion and Coleman 2025) or under longer‐term climate change scenarios. These programs can utilize environmental indicators like sea surface temperature, thermal thresholds, and current trajectories to forecast where tropical vagrants are likely to appear and assess the likelihood of their permanent establishment based on known physiological traits. This approach creates capacity to enable timely and targeted policy responses when required.

Experimental designs can play a critical role in refining predictions and informing management practices by identifying the specific traits that facilitate the successful establishment of tropical vagrant fishes. Traits such as thermal tolerance, reproductive strategies, habitat plasticity, and trophic interactions are likely to be key determinants of the potential for vagrant species to establish. For example, the expression of specific heat‐shock proteins (e.g., hsp70‐2) and the control they have over critical thermal limits/thermal niches contribute to latitudinal range shifts of coastal fishes (Fangue et al. 2006; Rincón‐Díaz et al. 2024). Controlled experiments that test the physiological thresholds of species traits, as well as their capacity for species to exploit novel food resources or adapt to local predator–prey dynamics, will shed light on the mechanisms supporting their survival in new habitats. Long‐term field studies that track vagrant species across environmental gradients can provide critical data on the conditions under which they transition from transient to resident populations (e.g., Booth et al. 2018). For example, in Canada the Salmon FIT‐CHIP program selects candidate genes whose increased expression may be useful to monitor long‐term thermal stress in local salmon populations living in thermally unstable habitats (i.e., vulnerable to climate warming) (Akbarzadeh et al. 2018). A similar approach could be applied in ecosystems experiencing increased vagrant presence, whereby monitoring programs non‐invasively measure (e.g., gill samples) the expression of key genes important for adapting to local conditions to determine long‐term persistence. By integrating experimental insights into predictive models that are used to produce management‐relevant information, policymakers can identify not only when tropical vagrants will arrive but also the tipping points at which they are likely to establish permanent populations (Champion et al. 2018) based on a suite of physiological traits. This trait‐based approach offers a powerful tool for managing the arrival of novel species in traditionally temperate marine ecosystems, as it enables early detection of high‐risk species and flags the need for proactive interventions. For example, a model conservation system to apply our trait‐based framework would be for marine protected area (MPA) establishment and maintenance, as these are vulnerable systems, especially for invasive species (Lima et al. 2021). Therefore, for these protected systems, it will be important to understand the suite of physiological traits that enhance acclimation and long‐term persistence of any vagrants, which species from adjacent habitats may move into MPAs based on selected traits to survive under MPA conditions, and the ramifications on MPA function associated with range‐extending vagrants that are able to survive long‐term. Ultimately, leveraging species traits has vast potential to support more targeted and resource‐efficient management of species that can inform the decision of whether to persecute, protect, or ignore range‐shifting species (Scheffers and Pecl 2019).

A critical path forward will require prioritization of potential biomarkers for monitoring and implantation into management, especially when resources and finances are limited. The first step would be to establish a suite of well‐studied traits that are directly impacted by cold temperature stress in vagrant fish across acute and chronic scales. The easiest starting point would be whole‐animal measures such as growth rates, oxygen uptake rates, and swimming performance, as these traits have been well studied in tropical fishes in temperate waters, provide repeatable and reliable results, and are cost‐effective. If resources are limited, whole‐animal metrics such as morphometrics, growth rates and indices of reproduction (e.g., gonadosomatic index) can be sourced from fisheries and monitoring programs, which typically have a wealth of information on local, target and bycatch species in the area and are a cost‐effective approach to understand population responses to environmental change across multiple species. Such a collaboration with fisheries and monitoring programs may also provide insight into which species are invading temperate ecosystems over time (i.e., first‐hand accounts of presence/absence; “Step 2” of the framework), and identify species of key ecological and/or economic importance strongly affected by climate change (e.g., temperature, pH, dissolved oxygen) that will likely be of primary significance and should be a focus priority. Next, partnering whole‐animal traits with well‐established techniques from basal levels of biological organization, such as stress hormones (e.g., cortisol) and a few select genes (e.g., metabolic pathway changes, energy storage) will provide a mechanistic viewpoint of predicting future success in new habitats of tropical species (Step 4, Table 1). Measuring the whole transcriptome is expensive, and therefore, a cost‐effective solution would be to target a few select genes (see Table 1) and measure their expression using qPCR. Results from measuring a suite of traits may help refine future assessments of which traits are valuable for vagrants to adapt to local conditions.

Once it has been established how selected traits strongly linked with temperature vary among species (vagrants and local species), this information is highly suitable for incorporation into climate change vulnerability assessment frameworks that integrate species exposure and sensitivity to environmental change (Champion et al. 2023). Specifically, knowledge of how physiological traits linked to temperature vary among species can be used to refine estimates of species sensitivities, and thus vulnerability, to climate change (Foden et al. 2019). For example, species associated with switching metabolic pathways under cold stress, dietary breadth, use of alternative energy stores, and production of antioxidants and increased lipid synthesis have been linked with enhanced capacity to withstand cool water events, relative to species associated with an inability to modify their physiology in a similar way, making these individuals more likely to successfully overwinter in high latitudes and thus progress through stages of climate‐driven redistributions (Bates et al. 2014). In these instances, vulnerability assessments that support the management of marine fishes under climate change may be refined by reducing the sensitivity of species associated with the aforementioned traits, given species characterized by these physiological traits appear more likely to persist and survive through stages of climate‐driven redistributions. It could then be concluded that once information on species physiological traits has been used to refine current climate change vulnerability assessments for fishes, ranking indices can be applied to prioritize species that are most vulnerable for development of, or inclusion within existing, climate adaptation management plans and strategies (Champion and Coleman 2025).

## 6 Conclusions

The impacts of tropicalization will continue to have widespread ecological, economic, and social ramifications globally. Moving forward, the integration of an expansive range of physiological traits that complement ecological methods will be critical in furthering our understanding of range expansion in marine systems. Here, we develop a framework highlighting the increased demand of integrating physiological traits for fishes, especially primary and secondary responses, to not only understand the fundamental mechanisms driving species range expansions but also lead the development of biomarkers with the application of monitoring species' success or failure in moving into temperate habitats. We outline key steps in experimental design researchers should consider for both lab and in situ studies that complement current practices but also move the field forward in a more cohesive way through the consideration of wider life‐history stages, sentinel species, and physiological traits spanning biological organization. Taken together, our approach extends beyond our case‐study system of Australia because the principles of thermal adaptation are similar globally for most fish species, and applying the proposed physiological traits would provide a more holistic perspective of why certain species are successful at range expansion and facilitate our ability to predict which species may move and be successful in new habitats.

## Author Contributions

**Adam T. Downie:** conceptualization, investigation, methodology, visualization, writing – original draft, writing – review and editing. **Curtis Champion:** conceptualization, investigation, methodology, writing – original draft, writing – review and editing. **David J. Booth:** conceptualization, investigation, methodology, supervision, writing – original draft, writing – review and editing.

## Conflicts of Interest

The authors declare no conflicts of interest.

## Data Availability

This review synthesizes information from the published literature and does not extract data from the literature or generate new data. Figures 3 and 4 adapt natural habitat range data on Indo‐Pacific Sergeant/Sergeant Major ( *Abudefduf vaigiensis* ) along the East coast of Australia from FishBase (Froese and Pauly 2025; sourced from Aquamaps 2019, Kaschner et al. 2019). Figure 4 adapts poleward range expansion of *A. vaigiensis* from natural ranges from RedMap Australia (RedMap Australia n.d., Pecl et al. 2019). Data provided by Redmap (redmap.org.au), a project initiated by the Institute for Marine and Antarctic Studies (IMAS), University of Tasmania, for monitoring of marine species range shifts operated within Australia by the University of Tasmania. https://www.redmap.org.au/species/1/3/. September 2024.
